# Lower extremity EMG-driven modeling of walking with automated adjustment of musculoskeletal geometry

**DOI:** 10.1371/journal.pone.0179698

**Published:** 2017-07-11

**Authors:** Andrew J. Meyer, Carolynn Patten, Benjamin J. Fregly

**Affiliations:** 1 Department of Mechanical & Aerospace Engineering, University of Florida, Gainesville, FL, United States of America; 2 Department of Physical Therapy, University of Florida, Gainesville, FL, United States of America; 3 Neural Control of Movement Lab, Malcom Randall VA Medical Center, Gainesville, FL, United States of America; University of Manchester, UNITED KINGDOM

## Abstract

Neuromusculoskeletal disorders affecting walking ability are often difficult to manage, in part due to limited understanding of how a patient’s lower extremity muscle excitations contribute to the patient’s lower extremity joint moments. To assist in the study of these disorders, researchers have developed electromyography (EMG) driven neuromusculoskeletal models utilizing scaled generic musculoskeletal geometry. While these models can predict individual muscle contributions to lower extremity joint moments during walking, the accuracy of the predictions can be hindered by errors in the scaled geometry. This study presents a novel EMG-driven modeling method that automatically adjusts surrogate representations of the patient’s musculoskeletal geometry to improve prediction of lower extremity joint moments during walking. In addition to commonly adjusted neuromusculoskeletal model parameters, the proposed method adjusts model parameters defining muscle-tendon lengths, velocities, and moment arms. We evaluated our EMG-driven modeling method using data collected from a high-functioning hemiparetic subject walking on an instrumented treadmill at speeds ranging from 0.4 to 0.8 m/s. EMG-driven model parameter values were calibrated to match inverse dynamic moments for five degrees of freedom in each leg while keeping musculoskeletal geometry close to that of an initial scaled musculoskeletal model. We found that our EMG-driven modeling method incorporating automated adjustment of musculoskeletal geometry predicted net joint moments during walking more accurately than did the same method without geometric adjustments. Geometric adjustments improved moment prediction errors by 25% on average and up to 52%, with the largest improvements occurring at the hip. Predicted adjustments to musculoskeletal geometry were comparable to errors reported in the literature between scaled generic geometric models and measurements made from imaging data. Our results demonstrate that with appropriate experimental data, joint moment predictions for walking generated by an EMG-driven model can be improved significantly when automated adjustment of musculoskeletal geometry is included in the model calibration process.

## Introduction

Neuromusculoskeletal disorders such as cerebral palsy [1], stroke [2], Parkinson’s disease [3], and osteoarthritis [4] hinder walking ability and decrease quality of life for millions of people. Rehabilitation treatments have been developed to attempt to improve the walking ability of individuals with these disorders. However, the effectiveness of these treatments can vary between patients, in part due to the use of treatment design methods based more on subjective than objective methods [5]. For instance, for medial knee osteoarthritis, recent studies using instrumented knee implants found that gait modifications expected to reduced medial knee contact force [6–8] did not always do so [9,10]. Similarly, stroke rehabilitation methods that are effective for some patients may be ineffective for others [11]. Thus, treatment outcomes for neuromusculoskeletal disorders could potentially be improved through the use of more objective treatment design methods.

To assist with the design of more effective interventions, researchers have developed neuromusculoskeletal models of individual patients. A major challenge in neuromusculoskeletal modeling is determining how muscles contribute to net joint moments. Some studies have used electromyography (EMG) data with [12–19] and without [20–24] geometric musculoskeletal models to estimate the joint moments generated by muscles during movement. When geometric models are used, EMG-driven models predict net joint moments in three steps. First, muscle activation is determined from EMG data using a first or second order activation dynamics model [12,25]. Next, muscle force is determined from muscle activation and muscle-tendon kinematics using Hill-type muscle models [26,27]. Finally, joint moments are determined by combining estimated muscle forces with calculated muscle moment arms, which requires geometric modeling of muscle-tendon origins, insertions, and lines of action around bones and other muscles. To reproduce experimental joint moments as closely as possible, researchers calibrate parameter values in the activation dynamics (e.g., activation and deactivation time constants, electromechanical delays) and Hill-type muscle (e.g., optimal muscle fiber lengths, tendon slack lengths, peak isometric strengths) models using optimization methods, where experimental joint moments are calculated via inverse dynamics assuming no uncertainty in experimental inputs (i.e., ground reactions, marker motions) or skeletal model parameter values (i.e., joint positions and orientations, segment mass properties) [12,14–16,28,29]. In contrast to the use of optimization, muscle-tendon kinematic and muscle moment arm information needed for the last two steps is typically provided by a scaled generic musculoskeletal model. However, several studies have demonstrated that scaled models may not represent the musculoskeletal geometry of individual subjects well [30–32]. Despite the presence of errors in muscle-tendon kinematics and moment arms in scaled geometric models, no study to date has attempted to adjust these quantities automatically to improve the prediction of net joint moments from EMG data.

This paper presents a novel EMG-driven modeling method that calibrates not only standard activation dynamics and muscle-tendon model parameter values but also non-standard geometric musculoskeletal model parameter values related to muscle-tendon lengths and moment arms to patient walking data. The method was developed and evaluated using instrumented treadmill walking data collected from a high-functioning hemiparetic subject walking at five different speeds. Surrogate models of muscle-tendon length, velocity, and moment arms for each muscle were fitted as functions of joint angles [33,34] to data sampled from a scaled generic OpenSim [35] musculoskeletal model [36]. Surrogate model parameter values, along with activation dynamics and Hill-type muscle-tendon model parameter values, were adjusted via optimization such that lower extremity joint moments calculated from the subject’s EMG data matched the subject’s inverse dynamic joint moments from walking as closely as possible. Parameter values were adjusted for 35 muscles in each leg of the subject’s model to match the hip flexion-extension (FE), hip adduction-abduction (AA), knee flexion-extension (FE), ankle plantar-dorsiflexion (PDF), and ankle inversion-eversion (IE) moments during treadmill walking. Calibrated EMG-driven models were evaluated by predicting joint moments for walking trials withheld from calibration, including trials performed at faster non-calibration walking speeds.

## Methods

### Experimental data

To support development and evaluation of our proposed EMG-driven modeling method, we collected experimental walking data from a single high-functioning hemiparetic male subject (age 79 years, LE Fugl-Meyer Motor Assessment 32/34 pts, right-sided hemiparesis, height 1.7 m, mass 80.5 kg). All experimental procedures were approved by the University of Florida Health Science Center Institutional Review Board (IRB-01), and the subject provided written informed consent prior to participation. Motion capture (Vicon Corp., Oxford, UK), ground reaction (Bertec Corp., Columbus, OH), and EMG (Motion Lab Systems, Baton Rouge, LA) data were collected simultaneously while the subject walked on a split-belt instrumented treadmill (Bertec Corp., Columbus, OH) at five different speeds: 0.4, 0.5, 0.6, 0.7, and 0.8 m/s which included his preferred speed of 0.5 m/s. Motion capture data were recorded at a frequency of 100 Hz, and analog data were recorded at a frequency of 1000 Hz. More than 50 gait cycles were recorded for each walking speed. A static standing trial was also collected. The motion capture data were obtained using a modified Cleveland clinic marker set with additional markers added to the feet [37]. Ground reaction and marker motion data were filtered at a variable cut-off frequency of 7/tf Hz, where tf is the period of the gait cycle being processed, using a fourth-order zero phase lag Butterworth filter [38]. This variable cut-off frequency would cause data collected at a normal walking speed to be filtered at approximately 6 Hz.

EMG data were collected and processed for 16 muscles in each leg. These data used a combination of surface and fine-wire electrodes. Electrodes were placed following the SENIAM convention for surface electrodes [39] and the Delagi et al. Anatomical Guide for the Electromyographer for fine wire electrodes [40]. Surface EMG data were collected for gluteus maximus and medius, semimembranosus, biceps femoris long head, rectus femoris, vastus medialis and lateralis, medial gastrocnemius, tibialis anterior, peroneus longus, and soleus. Fine-wire EMG data were collected for adductor longus, iliopsoas, tibialis posterior, flexor digitorum longus, and extensor digitorum longus. EMG data were high-pass filtered at 40 Hz [12], demeaned, rectified, and then low-pass filtered at a variable cut-off frequency 3.5/tf Hz. Filtering was performed using a fourth-order zero phase lag Butterworth filter. EMG data from each muscle were normalized to the maximum value over all trials and resampled to 101 time points per gait cycle while keeping an additional 20 time points before the start of the cycle to permit modeling of electromechanical delay. In addition, each processed EMG signal was offset on a cycle-by-cycle basis so that the minimum value was zero.

### Model description

**Fig 1:**
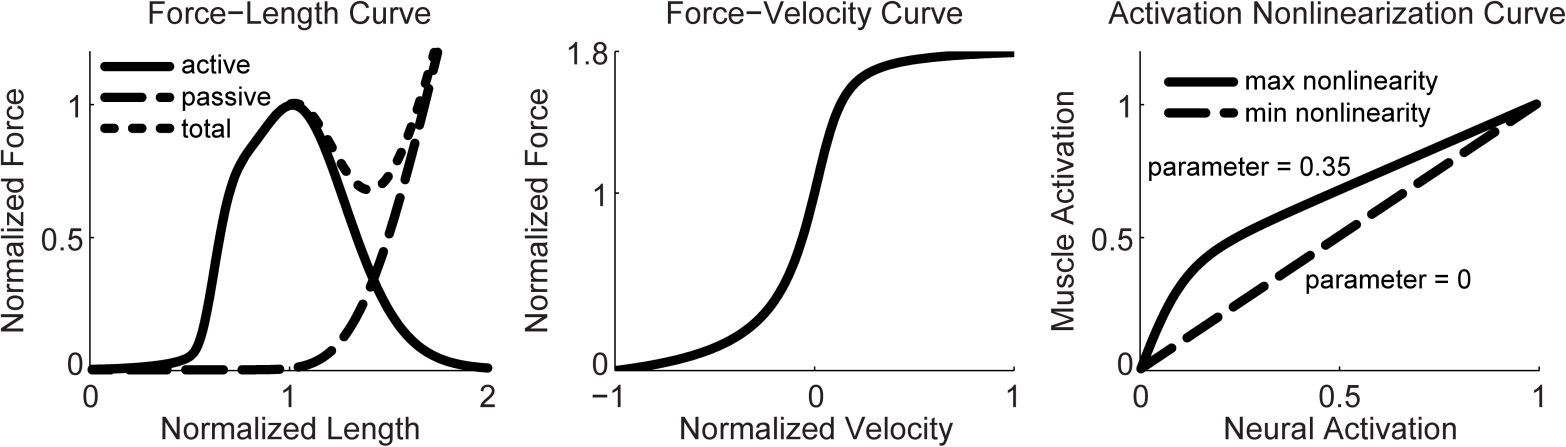
Relevant curves for our Hill-type muscle and activation nonlinearization model. Left: Normalized active, passive, and total force-length curves. Middle: Normalized force-velocity curve. Right: Neural-to-muscle activation nonlinearization curves for minimum nonlinearization (curve parameter = 0) and maximum nonlinearization (curve parameter = 0.35).

Our EMG-driven model uses a Hill-type muscle model with a rigid tendon [41] along with automatically adjusted musculoskeletal geometry. However, the necessary muscle-tendon lengths, velocities, and moment arms commonly obtained from a geometric musculoskeletal model are instead approximated by polynomial functions of model generalized coordinates and their first derivatives [33,34]. Each muscle’s moment about a spanned joint is represented by the following equation:

$$\begin{array}{c} M=r\cdot F_{o}^{M}\cdot \left[a(e(t-d))\cdot f_{l}({\tilde{l}}^{M}(t))\cdot f_{v}({\tilde{v}}^{M}(t))+f_{p}({\tilde{l}}^{M}(t))\right]\cos\alpha \\ 0<a(t)<1\\ 0.3<{\tilde{l}}^{M}(t)<1.3\\ -1<{\tilde{v}}^{M}(t)<1 \end{array}$$ (1)

where *M* is the moment about a given joint produced by the muscle, *r* is the moment arm of the muscle about the spanned joint, $F_{o}^{M}$ is the peak isometric force of the muscle, *a* is the muscle’s activation which is a function of processed experimental EMG data *e*, *t* is time, *d* is an electromechanical time delay, ${\tilde{l}}^{M}$ and ${\tilde{v}}^{M}$ are the normalized muscle fiber length and velocity, respectively, and *α* is the muscle pennation angle, which is assumed to remain constant to facilitate subsequent calibration of musculoskeletal geometry. Neglecting tendon compliance, ${\tilde{l}}^{M}$ and ${\tilde{v}}^{M}$ are calculated using the following equations:

$${\tilde{l}}^{M}=\frac{l^{MT}-l_{s}^{T}}{l_{o}^{M}\cos\alpha }$$ (2)

$${\tilde{v}}^{M}=\frac{v^{MT}}{10\cdot l_{o}^{M}}$$ (3)

where $l^{MT}$ is muscle-tendon length, $l_{o}^{M}$ is the optimal fiber length, $l_{s}^{T}$ is the tendon slack length, and *v*^*MT*^ is muscle-tendon velocity. $f_{l}({\tilde{l}}^{M}(t))$, $f_{p}({\tilde{l}}^{M}(t))$, and $f_{v}({\tilde{v}}^{M}(t))$ represent the normalized muscle active force-length, passive force-length, and force-velocity curves (Fig 1). In all, our Hill-type muscle model requires specification of five parameter values *d*, $l_{o}^{M}$, $l_{s}^{T}$, $F_{o}^{M}$, and *α* and four time varying quantities *a*, $l^{MT}$, *v*^*MT*^, and *r*. Methods for calculating these four time varying quantities are described below.

Muscle activation is calculated using a first order differential equation that describes excitation *e* to neural activation *u* dynamics and a nonlinear function that describes neural activation *u* to muscle activation *a* [42]. Neural activation is calculated by solving the first order differential equation proposed by He *et al*. [25]:

$$\frac{du(t)}{dt}=(c_{1}e(t-d)+c_{2})(e(t-d)-u(t))$$ (4)

where *e*(*t*−*d*) is excitation (i.e., processed EMG data) and *u*(*t*) is neural activation. The constants *c*_1_ and *c*_2_ are defined as:

$$c_{1}=\frac{1}{\tau_{act}}-\frac{1}{\tau_{deact}}$$ (5)

$$c_{2}=\frac{1}{\tau_{deact}}$$ (6)

where *τ*_*act*_ and *τ*_*deact*_ are muscle activation and deactivation time constants, respectively. These time constants are constrained to be proportional to each other such that *τ*_*deact*_ = 4*τ*_*act*_ based on the ratio commonly reported in the literature [27,43–45]. This linear differential equation is solved recursively over all time frames by discretizing Eq (4) at each time point using a high accuracy backward finite difference approximation, assuming neural activation at the first two time points equals time-delayed muscle excitation at these time points, and solving for the unknown neural activation at the current time point:

$$u_{i}=\frac{2\Delta t(c_{1}e(t_{i}-d)+c_{2})e(t_{i}-d)+4u_{i-1}-u_{i-2}}{2\Delta t(c_{1}e(t_{i}-d)+c_{2})+3}$$ (7)

where Δ*t* is the selected time interval and *i* represents the time frame for which neural activation is to be found. The nonlinear relationship between neural activation *u* and muscle activation *a* at time frame *i* is modeled using the equation:

$$a_{i}=(1-c_{3})u_{i}+c_{3}\left[\frac{g_{1}}{g_{2}{\left(u_{i}+g_{3}\right)}^{g_{4}}+g_{5}}+1\right]$$ (8)

where *c*_3_ is a constant that can vary from 0 (linear) to 0.35 (highly nonlinear), and *g*_1_-*g*_5_ are values determined by fitting published experimental data from isometric contractions [42]. Constant coefficients *g*_1_-*g*_5_ have values of -7.623, 29.280, 0.884, 17.227, and 4.108. This activation nonlinearity equation is a simplified version of functions proposed previously [42].

The time varying quantities $l^{MT}$, *v*^*MT*^, and *r* are calculated using polynomial functions of the joint angles and velocities that share common coefficients [33,34]. For muscles that span a single degree of freedom (DOF), the muscle-tendon length is approximated using the cubic polynomial equation:

$$l^{MT}(t)=b_{0}+b_{1}\theta +b_{2}\theta^{2}+b_{3}\theta^{3}$$ (9)

where $l^{MT}$ is muscle-tendon length, *θ* is joint angle, and *b*_0_ through *b*_3_ are constant coefficients. Muscle-tendon velocity *v*^*MT*^ can then be calculated using the first derivative with respect to time of Eq (9):

$$v^{MT}(t)=\frac{dl^{MT}}{dt}=b_{1}\dot{\theta }+2b_{2}\theta \dot{\theta }+3b_{3}\theta^{2}\dot{\theta }$$ (10)

where $\dot{\theta }$ is the joint angular velocity. Similarly, the muscle-tendon moment arm can be calculated from Eq (9) using a relationship from An et al. [46]:

$$r(t)=-\frac{\partial l^{MT}}{\partial \theta }=-b_{1}-2b_{2}\theta -3b_{3}\theta^{2}$$ (11)

The negative sign in this expression is needed for consistency with the OpenSim musculoskeletal modeling environment, where a positive joint moment causes a positive change in joint angle. For muscles that span two DOFs, these equations are extended as follows:

$$l^{MT}=b_{0}+b_{1}\theta_{1}+b_{2}\theta_{2}+b_{3}\theta_{1}\theta_{2}+b_{4}\theta_{1}^{2}+b_{5}\theta_{2}^{2}+b_{6}\theta_{1}^{2}\theta_{2}+b_{7}\theta_{1}\theta_{2}^{2}+b_{8}\theta_{1}^{3}+b_{9}\theta_{2}^{3}$$ (12)

$$\begin{array}{l} v^{MT}=b_{1}{\dot{\theta }}_{1}+b_{2}{\dot{\theta }}_{2}+b_{3}({\dot{\theta }}_{1}\theta_{2}+\theta_{1}{\dot{\theta }}_{2})+2b_{4}\theta_{1}{\dot{\theta }}_{1}+2b_{5}\theta_{2}{\dot{\theta }}_{2}+\ldots \\ \;b_{6}(2\theta_{1}{\dot{\theta }}_{1}\theta_{2}+\theta_{1}^{2}{\dot{\theta }}_{2})+b_{7}({\dot{\theta }}_{1}\theta_{2}^{2}+2\theta_{1}\theta_{2}{\dot{\theta }}_{2})+3b_{8}\theta_{1}^{2}{\dot{\theta }}_{1}+3b_{9}\theta_{2}^{2}{\dot{\theta }}_{2} \end{array}$$ (13)

$$r_{1}=-\frac{\partial l^{MT}}{\partial \theta_{1}}=-b_{1}-b_{3}\theta_{2}-2b_{4}\theta_{1}-2b_{6}\theta_{1}\theta_{2}-b_{7}\theta_{2}^{2}-3b_{8}\theta_{1}^{2}$$ (14)

$$r_{2}=-\frac{\partial l^{MT}}{\partial \theta_{2}}=-b_{2}-b_{3}\theta_{1}-2b_{5}\theta_{2}-b_{6}\theta_{1}^{2}-2b_{7}\theta_{1}\theta_{2}-3b_{9}\theta_{2}^{2}$$ (15)

For muscles that span three or four DOFs, Eqs (9), (10) and (11) are extended in a similar manner by adding terms corresponding to the additional joint angles and velocities. These polynomial functions can be viewed as surrogate models of muscle-tendon lengths, velocities, and moment arms.

### Model calibration

In our EMG-driven model calibration process, we start with a generic full-body OpenSim musculoskeletal model [35]. The authors of that study created this initial model using measurements made on 21 cadaveric specimens. Since the present study focuses on lower limb motion during walking, the generic model was reduced to 29 DOFs by removing toes, forearm, and wrist DOFs. The lower extremity joints were modeled as follows: the hips as ball-and-socket joints, the knees as hinge joints (flexion/extension) with prescribed translations defined as a function of knee rotation [47], and the ankles as two non-intersecting hinge joints. After removal of muscles without related EMG signals, 35 muscles remained whose names, functions, and excitation groups are listed in Table 1. Many of these muscles represented compartments of larger muscles that were split to model their function more accurately. For instance, gluteus maximus was split into three compartments modeled as individual muscles with a common excitation signal.

**Table 1:** List of muscles in the model, which DOF each muscle actuates, and source of each muscle’s excitation signal.

Muscle	Actuates	EMG Signal Source	EMG Type
**Adductor brevis**	Hip FE, Hip AA	Adductor longus	Fine wire
**Adductor longus**	Hip FE, Hip AA		
**Adductor magnus distal**	Hip FE, Hip AA		
**Adductor magnus ischial**	Hip FE, Hip AA		
**Adductor magnus middle**	Hip FE, Hip AA		
**Adductor magnus proximal**	Hip FE, Hip AA		
**Gluteus maximus superior**	Hip FE, Hip AA	Gluteus maximus	Surface
**Gluteus maximus middle**	Hip FE, Hip AA		
**Gluteus maximus inferior**	Hip FE, Hip AA		
**Gluteus medius anterior**	Hip FE, Hip AA	Gluteus medius	Surface
**Gluteus medius middle**	Hip FE, Hip AA		
**Gluteus medius posterior**	Hip FE, Hip AA		
**Gluteus minimus anterior**	Hip FE, Hip AA		
**Gluteus minimus middle**	Hip FE, Hip AA		
**Gluteus minimus posterior**	Hip FE, Hip AA		
**Iliacus**	Hip FE, Hip AA	Iliacus or Psoas	Fine wire
**Psoas**	Hip FE, Hip AA		
**Semimembranosus**	Hip FE, Hip AA, Knee FE	Semimembranosus	Surface
**Semitendinosus**	Hip FE, Hip AA, Knee FE		
**Biceps femoris long head**	Hip FE, Hip AA, Knee FE	Biceps femoris long head	Surface
**Biceps femoris short head**	Knee FE		
**Rectus femoris**	Hip FE, Hip AA, Knee FE	Rectus femoris	Surface
**Vastus medialis**	Knee FE	Vastus medialis	Surface
**Vastus intermedius**	Knee FE		
**Vastus lateralis**	Knee FE	Vastus lateralis	Surface
**Lateral gastrocnemius**	Knee FE, Ankle PDF, Ankle IE	Medial gastrocnemius	Surface
**Medial gastrocnemius**	Knee FE, Ankle PDF, Ankle IE		
**Tibialis anterior**	Ankle PDF, Ankle IE	Tibialis anterior	Surface
**Tibialis posterior**	Ankle PDF, Ankle IE	Tibialis posterior	Fine wire
**Peroneus brevis**	Ankle PDF, Ankle IE	Peroneus longus	Surface
**Peroneus longus**	Ankle PDF, Ankle IE		
**Peroneus tertius**	Ankle PDF, Ankle IE		
**Soleus**	Ankle PDF, Ankle IE	Soleus	Surface
**Extensor digitorum longus**	Ankle PDF, Ankle IE	Extensor digitorum longus	Fine wire
**Flexor digitorum Longus**	Ankle PDF, Ankle IE	Flexor digitorum longus	Fine wire

The first step in our EMG-driven model calibration process was scaling of the generic musculoskeletal model in OpenSim to match static trial marker data. Each segment’s scale factors were based on the ratio of distances between markers placed over bony landmarks and distances between corresponding markers in the generic model. Symmetry was maintained between the right and left sides of the body. The following segments were scaled: pelvis, torso, upper arms, forearms, thighs, shanks, and feet.

Following scaling, the next step was calibration of lower extremity joint positions and orientations and of marker positions within the body segments such that an OpenSim inverse kinematics analysis matched measured marker locations during walking as closely as possible [37,48]. This calibration step was performed in MATLAB (MathWorks, Natick, MA) via nonlinear least squares optimization and the OpenSim MATLAB application programming interface [35] for performing repeated inverse kinematic analyses. A single representative walking trial at 0.5 m/s, the subject’s preferred walking speed, was used for this purpose. Distances between pairs of markers within the same body segment were fixed during calibration. Joint positions and orientations within the body segments were adjusted only for the lower extremities while marker positions within the body segments were adjusted for all segments except the arms. Since relocating joint centers causes segment lengths to change, the model geometry was rescaled based on the new joint-to-joint distances. Model symmetry was maintained between the right and left sides during this calibration step.

Given the scaled musculoskeletal model with calibrated joint parameters, the third step of the calibration process was creation of surrogate models of muscle-tendon geometry using Eqs (9–15) [33,34]. Each muscle’s muscle-tendon length and moment arms were calculated by OpenSim [49] for 1000 different model poses specified using Latin hypercube sampling over a wide range of joint angles that went well beyond those that occur during walking. Surrogate models of muscle-tendon lengths and moment arms were then fitted simultaneously by calculating model coefficients using linear least squares regression. Muscle-tendon velocities were not matched because the sampling process was time independent. The resulting surrogate geometric models closely reproduced the subject’s muscle-tendon lengths, velocities, and moment arms for walking as calculated by the scaled OpenSim musculoskeletal model with calibrated joint parameters. Median fitting errors for all muscles were less than 1.6 mm for moment arms and 0.69 mm for muscle-tendon lengths.

**Fig 2:**
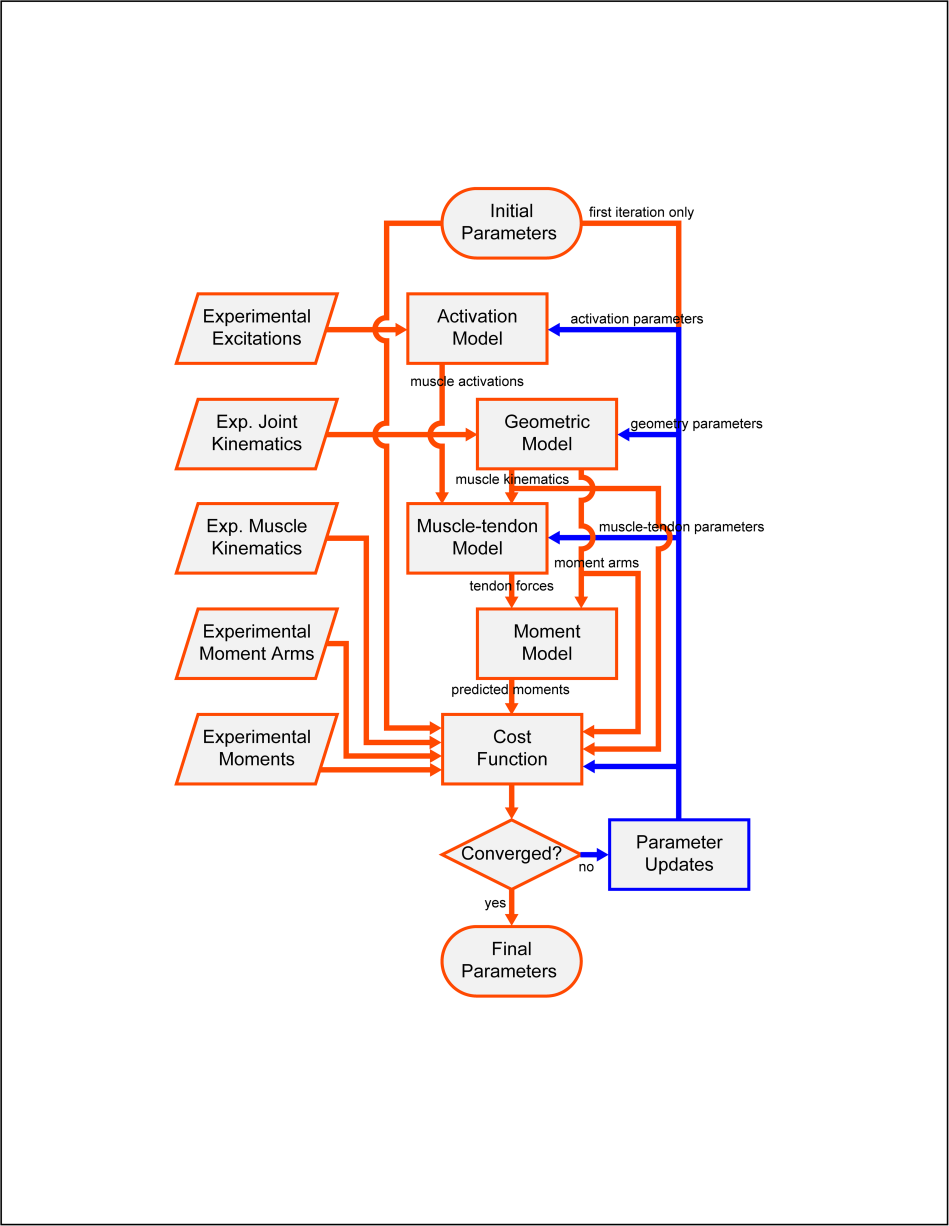
Flowchart of EMG-driven model calibration process for walking. The goal is to find model parameter values (i.e., activation parameters, surrogate geometry parameters, and muscle-tendon parameters) such that experimental processed EMG data and joint kinematics can be input to the model and lower extremity joint moments that closely match experimental joint moments are output from the model. Blue lines indicate model parameter values changed by the optimization process.

The final step of the calibration process was creation of an EMG-driven model by optimizing activation, Hill-type muscle-tendon, and surrogate geometric model parameter values for all muscles such that lower extremity joint moments predicted by the model matched those calculated by inverse dynamics as closely as possible (Fig 2). Because of the large number of design variables and quantities being tracked in the cost function, the optimization was highly over-constrained. The design variables altered by the optimization were: electromechanical delays *d*, activation time constants *τ*_*act*_, activation nonlinearity constants *c*_3_, scale factors defining the maximum processed EMG value achievable by each muscle, common scale factors for the optimal muscle fiber length and tendon slack length of each muscle, and coefficients *b*_0_ through *b*_*n*_ defining muscle-tendon lengths, velocities, and moment arms. These model parameter values were calibrated using a sequence of seven optimizations to reduce the likelihood of entrapment in a local minimum. In the first and fourth optimizations, electromechanical delays, muscle activation time constants, activation nonlinearity constants, and EMG scale factors were adjusted while all other design variables were fixed at their initial or previous values. In the second and fifth optimizations, common scale factors for optimal muscle fiber lengths and tendon slack lengths were adjusted. In the third and sixth optimizations, coefficients defining muscle-tendon geometry were adjusted. Finally, in the seventh optimization, all design variables were adjusted simultaneously. All optimizations were performed using MATLAB’s fmincon sequential quadratic programming algorithm [50].

To maintain anatomic realism, the cost function for these optimizations not only minimized errors in model-predicted lower extremity joint moments but also penalized changes in model parameter values, muscle kinematics, and muscle moment arms away from their initial values and trajectories [48]. Joint moment errors were calculated for both active and passive moments. Active moments were calculated from the subject’s walking data via an OpenSim inverse dynamic analysis performed for ten gait cycles from each walking speed. Passive moments were taken from measurements reported in the literature for a wide range of joint angle combinations [51]. These passive moment data were included to provide additional information for estimating passive muscle-tendon properties. Initial model parameter values were either taken directly from the literature or customized to the subject based on information in the literature (for example, peak isometric force values were calculated using information reported in [52]), while initial muscle kinematic and moment arm trajectories were taken from the subject’s scaled OpenSim model. Details regarding specification of initial guesses, variable bounds, and cost function terms can be found in the S1 Appendix.

### Model evaluation

Using the optimization process described above, we evaluated our EMG-driven modeling process by performing two “calibrate, then test” scenarios. Gait cycles from all walking speeds were selected for this process. To develop the necessary inputs for calibration and testing, we performed OpenSim inverse kinematic and inverse dynamic analyses for each walking cycle. Using the inverse kinematic results, we generated reference muscle-tendon length, velocity, and moment arm curves from the surrogate geometric models, which avoided potential discontinuities caused by problems associated with muscle wrapping surfaces. All EMG, inverse kinematic, inverse dynamic, and muscle-tendon geometric curves were resampled to 101 time points per walking cycle. In addition, to prevent numerical issues at heel strike and toe off, and to accommodate identification of electromechanical delays, we included 20 additional time frames of all data before the start of each gait cycle. Given the curves output by OpenSim analyses, we identified and removed outlier trials using criteria described in the S1 Appendix.

The two “calibrate, then test” evaluation scenarios differed based on whether or not the testing phase included walking data from faster speeds not included in the calibration phase. For the first scenario, model calibration was performed using 50 trials of data from all five walking speeds (10 trials per speed) and model testing was performed using an additional 50 trials of data from the same five speeds. For the second scenario, model calibration was performed using 30 trials of data from the three slowest walking speeds (10 trials per speed) and model testing was performing using an additional 50 trials of data from all five walking speeds, including 0.7 and 0.8 m/s. For both scenarios, two EMG-driven models, one with and one without geometric adjustments, were calibrated via optimization to match inverse dynamic joint moment data from the calibration walking trials. All models were adjusted to match joint moments for five DOFs in each leg: hip flexion extension, hip adduction-abduction, knee flexion-extension, ankle plantar-dorsiflexion, and ankle inversion-eversion. Since EMG data were collected from only 16 muscles in each leg, excitations for muscles without EMG data were specified using EMG data from related muscles [14]. A list of the muscles used in the model, the associated joints they actuate, and the EMG signals that control them can be found in Table 1. Using only joint kinematics and processed EMG signals as inputs, the calibrated EMG-driven models were used to predict joint moments at each speed for 10 walking trials withheld from calibration. Mean absolute errors (MAE) between predicted and inverse dynamic joint moments were calculated across each gait cycle to evaluate the accuracy of all EMG-driven models:

$$\mathrm{MAE}=\frac{1}{n}\sum_{i=1}^{n}\left|M_{i}^{ID}-M_{i}^{EtM}\right|$$ (16)

where $M_{i}^{ID}$ is a moment from inverse dynamics, $M_{i}^{EtM}$ is the corresponding moment predicted by an EMG-driven model, and *n* is the number of time frames being evaluated. For each speed-joint-side combination, we performed a non-parametric Wilcoxon signed-rank test to evaluate whether the 10 MAE values for the two methods were statistically different. For average MAE differences across all speeds for each joint-side combination, we performed a non-parametric Friedman’s test with blocking based on gait speed. For all statistical tests, the level of statistical significance was set at *p* < 0.05.

## Results

**Fig 3:**
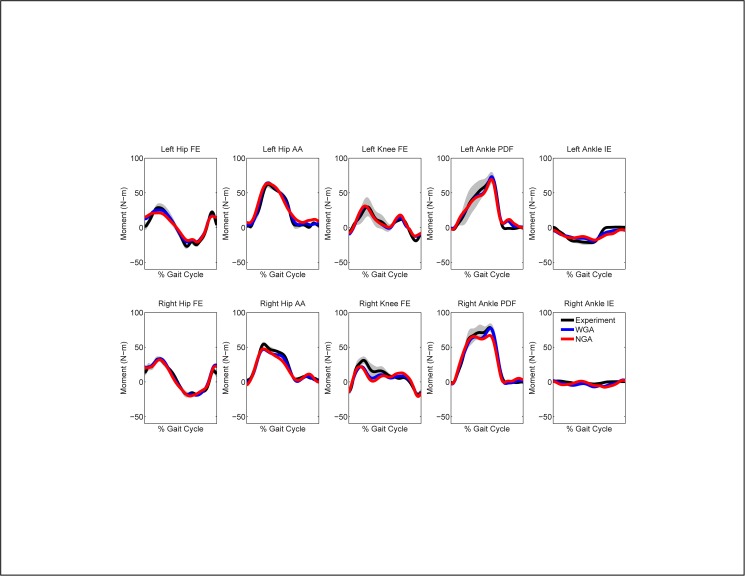
Average joint moment predictions for walking at 0.5 m/s when calibrating using all five walking speeds. NGA stands for no geometric adjustments and WGA stands for with geometric adjustments. Average experimental values with gray bands specifying +/- 1 standard deviation are shown for visualization purposes and were calculated at each time point after each gait cycle was resampled to 101 points.

**Fig 4:**
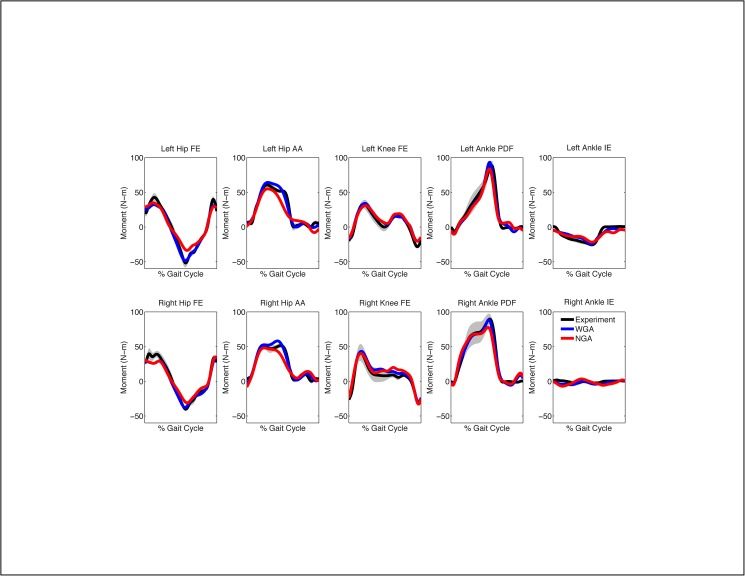
Average joint moment predictions for walking at 0.8 m/s when calibrating using all five walking speeds. NGA stands for no geometric adjustments and WGA stands for with geometric adjustments. Average experimental values with gray bands specifying +/- 1 standard deviation are shown for visualization purposes and were calculated at each time point after each gait cycle was resampled to 101 points.

When calibrated using walking data from all five speeds, the EMG-driven model with geometric adjustments (henceforth the “WGA model”) produced more accurate moment predictions for all joints than did the model without geometric adjustments (henceforth the “NGA model”) (Figs 3 and 4, Table 2). For additional walking trials not used in the calibration process, geometric adjustments improved joint moment predictions by an average of 25%, with the largest improvements occurring at the hip (33%), following by the ankle (21%), and finally the knee (16%). The largest average improvement for any joint moment occurred for left hip adduction-abduction (43%). Improvements produced by adding geometric adjustments were generally comparable between legs and across walking speeds. From a statistical standpoint, 85% of the calculated percent changes in MAE were statically significant, with only the right ankle inverse-eversion moment demonstrating few statistically significant changes.

**Table 2:** Mean MAE values for testing trials using EMG-driven models calibrated at all available walking speeds without (NGA) and with (WGA) geometric adjustments. The percent change in MAE when geometric adjustments were added is also reported, with the standard deviation of MAE between trials shown in parenthesis.

Gait Speed	Model Type	Hip FE (N-m)		Hip AA (N-m)		Knee FE (N-m)		Ankle PDF (N-m)		Ankle IE (N-m)	
		Right	Left	Right	Left	Right	Left	Right	Left	Right	Left
0.4 m/s	NGA	5.28 (1.34)	5.31 (1.39)	7.23 (0.83)	7.91 (1.57)	6.6 (0.49)	5.43 (1.26)	7.44 (2.29)	5.96 (1.08)	2.48 (1.46)	6.18 (1.14)
	WGA	3.83 (0.81)	4.61 (1.29)	3.58 (0.46)	4.99 (1.03)	5.22 (0.66)	4.69 (0.94)	5.44 (2.66)	5.28 (0.98)	2.27 (1.85)	4.88 (0.34)
	% Change	-27.43*	-13.19	-50.46*	-36.92*	-20.93*	-13.71*	-26.96*	-11.43	-8.54	-21.09*
0.5 m/s	NGA	4.23 (1.03)	5.94 (1.28)	5.87 (0.72)	7.64 (2.11)	7.07 (0.78)	4.67 (1.18)	7.50 (2.15)	7.14 (0.89)	3.52 (2.47)	5.74 (0.73)
	WGA	3.47 (0.64)	4.47 (1.23)	4.02 (1.36)	4.26 (1.69)	5.33 (0.64)	4.28 (0.83)	4.85 (0.94)	6.09 (1.16)	3.46 (2.74)	4.40 (0.91)
	% Change	-18.06*	-24.69*	-31.49*	-44.30*	-24.62*	-8.26	-35.31*	-14.70*	-1.90	-23.43*
0.6 m/s	NGA	5.46 (1.43)	6.76 (0.89)	8.01 (1.89)	7.81 (0.86)	5.33 (1.14)	5.11 (1.49)	7.27 (1.92)	7.24 (2.39)	3.42 (1.15)	5.98 (1.19)
	WGA	4.10 (0.75)	4.94 (1.33)	4.55 (0.60)	4.46 (0.73)	4.27 (0.85)	4.81 (1.50)	4.89 (2.03)	5.63 (0.75)	2.84 (1.40)	4.27 (0.93)
	% Change	-24.91*	-27.00*	-43.23*	-42.92*	-19.91*	-5.78	-32.76*	-22.21*	-17.04*	-28.53*
0.7 m/s	NGA	6.33 (1.05)	7.08 (0.94)	6.87 (1.37)	8.26 (1.09)	5.52 (1.07)	6.05 (1.58)	8.01 (2.33)	7.43 (1.52)	3.15 (1.31)	5.78 (1.04)
	WGA	4.73 (1.10)	5.50 (0.99)	4.41 (1.48)	4.46 (1.30)	4.55 (0.52)	4.92 (1.52)	5.98 (1.33)	5.51 (0.91)	2.95 (1.33)	4.05 (0.64)
	% Change	-25.34*	-22.22*	-35.88*	-45.98*	-17.50	-18.58*	-25.34*	-25.85*	-6.23	-30.02*
0.8 m/s	NGA	6.92 (0.79)	7.45 (1.03)	7.17 (1.06)	8.81 (1.70)	6.43 (0.61)	4.76 (1.12)	8.48 (1.79)	7.24 (1.26)	3.56 (1.73)	5.50 (0.54)
	WGA	5.11 (1.30)	5.04 (1.10)	4.55 (0.62)	4.79 (0.98)	5.28 (0.56)	4.22 (1.39)	5.93 (1.56)	5.99 (1.18)	2.91 (1.41)	4.22 (0.39)
	% Change	-26.17*	-32.32*	-36.50*	-45.61*	-17.90*	-11.31*	-30.11*	-17.29*	-18.15*	-23.38*
Average	NGA	5.65	6.51	7.03	8.09	6.19	5.20	7.74	7.00	3.23	5.84
	WGA	4.25	4.91	4.22	4.59	4.93	4.58	5.42	5.70	2.88	4.36
	% Change	-24.76*	-24.50*	-39.95*	-43.22*	-20.36*	-11.87*	-30.02*	-18.60*	-10.56	-25.27*

* indicates a statistically significant change (*p* < 0.05) based on Wilcoxon signed-rank tests. For average differences, a Friedman’s test with blocking based on gait speed was used.

**Fig 5:**
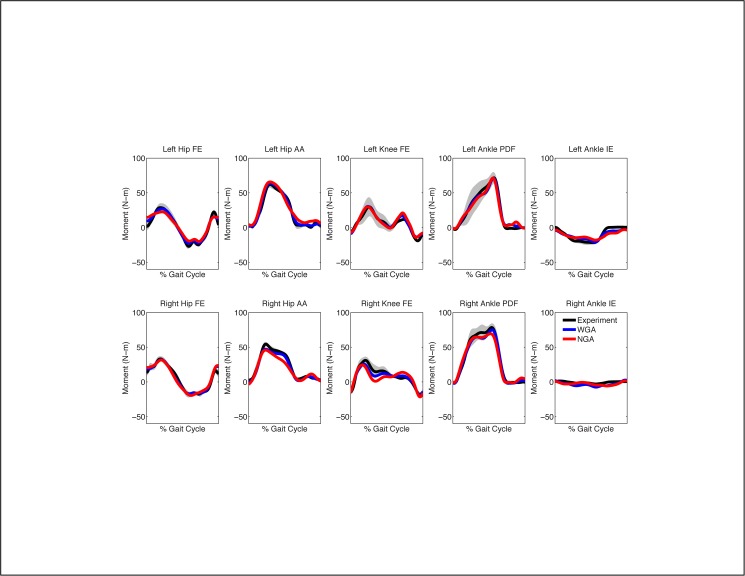
Average joint moment predictions for walking at 0.5 m/s when calibrating using only the three slowest walking speeds. NGA stands for no geometric adjustments and WGA stands for with geometric adjustments. Average experimental values with gray bands specifying +/- 1 standard deviation are shown for visualization purposes and were calculated at each time point after each gait cycle was resampled to 101 points.

**Fig 6:**
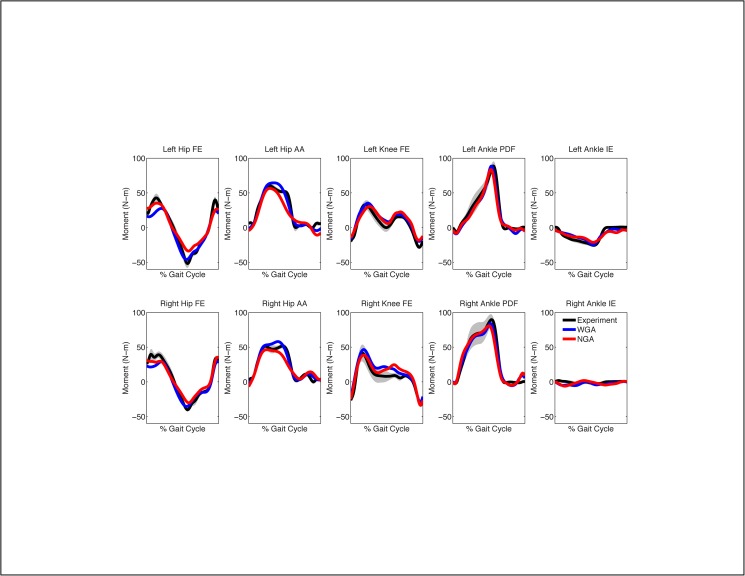
Average joint moment predictions for walking at 0.8 m/s when calibrating using only the three slowest walking speeds. NGA stands for no geometric adjustments and WGA stands for with geometric adjustments. Average experimental values with gray bands specifying +/- 1 standard deviation are shown for visualization purposes and were calculated at each time point after each gait cycle was resampled to 101 points.

When calibrated using walking data from only the three slowest speeds, the WGA model again produced more accurate moment predictions for all joints than did the NGA model, with the one exception being the right ankle inversion-eversion moment (Figs 5 and 6, Table 3). For additional walking trials at speeds used in the calibration process, geometric adjustments improved joint moment predictions by an average of 23%, with the largest improvements occurring at the hip (34%), following by the knee (22%), and finally the ankle (12%). The largest average improvement for any joint moment occurred for right hip adduction-abduction (46%). For additional walking trials at faster speeds not used for calibration, geometric adjustments improved joint moment predictions by an average of 15%, with the largest improvements again occurring at the hip (23%), following by the ankle (10%), and finally the knee (9%). The largest average improvement for any joint moment again occurred for left hip adduction-abduction (37%). As noted above, the one exception was the right ankle inversion-eversion moment, which exhibited worse moment predictions (9% of an extremely small moment) with the addition of geometric adjustments. From a statistical standpoint, 72% of the calculated percent changes in MAE were statically significant, with only the right ankle inversion-eversion and left ankle plantarflexion-dorsiflexion moments demonstrating few statistically significant changes.

**Table 3:** Mean MAE values for testing trials using EMG-driven models calibrated at 0.4, 0.5, and 0.6 m/s walking speeds without (NGA) and with (WGA) geometric adjustments. The percent change in MAE when geometric adjustments were added is also reported, with the standard deviation of MAE between trials shown in parenthesis. The bold row headers indicate the gait speeds being predicted that were not included in model calibration.

Gait Speed	Model Type	Hip FE (N-m)		Hip AA (N-m)		Knee FE (N-m)		Ankle PDF (N-m)		Ankle IE (N-m)	
		Right	Left	Right	Left	Right	Left	Right	Left	Right	Left
0.4 m/s	NGA	5.06 (1.39)	5.13 (1.48)	6.98 (0.70)	7.77 (1.31)	6.69 (0.50)	5.34 (1.34)	7.25 (2.39)	5.06 (1.10)	2.01 (1.21)	5.82 (1.03)
	WGA	3.78 (0.73)	4.45 (1.00)	3.37 (0.55)	4.99 (1.22)	4.37 (0.57)	4.50 (0.86)	5.79 (2.37)	4.80 (1.34)	2.07 (1.78)	4.60 (0.40)
	% Change	-25.21*	-13.30	-51.67*	-35.77*	-34.68*	-15.69*	-20.22*	-5.08	2.96	-20.88*
0.5 m/s	NGA	4.32 (1.09)	5.77 (1.45)	6.41 (0.94)	7.38 (1.78)	7.24 (0.74)	4.47 (1.22)	6.8 (2.25)	6.02 (1.09)	2.88 (2.04)	5.29 (0.59)
	WGA	3.15 (0.73)	4.20 (0.97)	3.57 (1.07)	4.21 (1.85)	4.18 (0.77)	3.93 (0.87)	4.89 (1.02)	5.56 (1.20)	3.50 (2.79)	4.00 (0.65)
	% Change	-26.96*	-27.25*	-44.28*	-42.92*	-42.26*	-12.12*	-28.07*	-7.72	21.55*	-24.41*
0.6 m/s	NGA	5.54 (1.52)	6.64 (0.89)	8.26 (2.38)	7.70 (0.92)	6.39 (1.37)	5.68 (1.46)	6.74 (1.82)	5.79 (2.53)	2.70 (0.90)	5.53 (1.04)
	WGA	4.30 (1.12)	4.76 (1.49)	4.71 (1.06)	4.39 (0.92)	5.88 (0.69)	4.84 (1.30)	5.23 (2.01)	5.30 (1.16)	2.70 (1.02)	3.83 (0.72)
	% Change	-22.43*	-28.41*	-42.95*	-43.00*	-7.97*	-14.77*	-22.41*	-8.47*	-0.14	-30.70*
Average	NGA	4.97	5.85	7.22	7.62	6.77	5.17	6.93	5.62	2.53	5.55
	WGA	3.75	4.47	3.89	4.53	4.81	4.43	5.30	5.22	2.76	4.14
	% Change	-24.68*	-23.61*	-46.16*	-40.51*	-28.99*	-14.32*	-23.49*	-7.19	8.90	-25.27*
**0.7 m/s**	NGA	6.50 (1.09)	7.38 (1.11)	7.42 (2.02)	8.13 (1.37)	6.26 (1.1)	7.27 (1.43)	7.66 (1.89)	6.45 (1.34)	2.46 (1.23)	5.29 (0.98)
	WGA	5.14 (1.04)	6.70 (1.18)	4.67 (2.02)	5.21 (1.5)	6.23 (0.62)	6.12 (1.50)	6.18 (1.30)	5.93 (0.93)	2.95 (1.35)	3.73 (0.47)
	% Change	-20.93*	-9.30*	-37.02*	-35.97*	-0.46	-15.84*	-19.29*	-8.01*	20.23*	-29.48*
**0.8 m/s**	NGA	7.02 (0.87)	7.42 (1.27)	7.50 (1.50)	9.40 (1.88)	7.75 (0.62)	6.75 (1.29)	8.03 (2.10)	6.63 (1.45)	2.95 (1.66)	5.19 (0.52)
	WGA	6.25 (1.61)	7.34 (1.20)	4.89 (1.01)	5.84 (0.88)	7.52 (0.57)	5.65 (1.53)	6.45 (1.63)	6.54 (1.98)	2.92 (1.21)	4.08 (0.32)
	% Change	-10.93	-1.06	-34.79*	-37.87*	-2.91	-16.35*	-19.74*	-1.41	-1.17	-21.32*
**Average**	NGA	6.76	7.40	7.46	8.76	7.01	7.01	7.85	6.54	2.70	5.24
	WGA	5.69	7.02	4.78	5.52	6.88	5.89	6.32	6.23	2.94	3.91
	% Change	-15.74*	-5.17	-35.90*	-36.99*	-1.81	-16.09*	-19.52*	-4.66	8.55	-25.44*

* indicates a statistically significant change (*p* < 0.05) based on Wilcoxon signed-rank tests. For average differences, a Friedman’s test with blocking based on gait speed was used.

**Fig 7:**
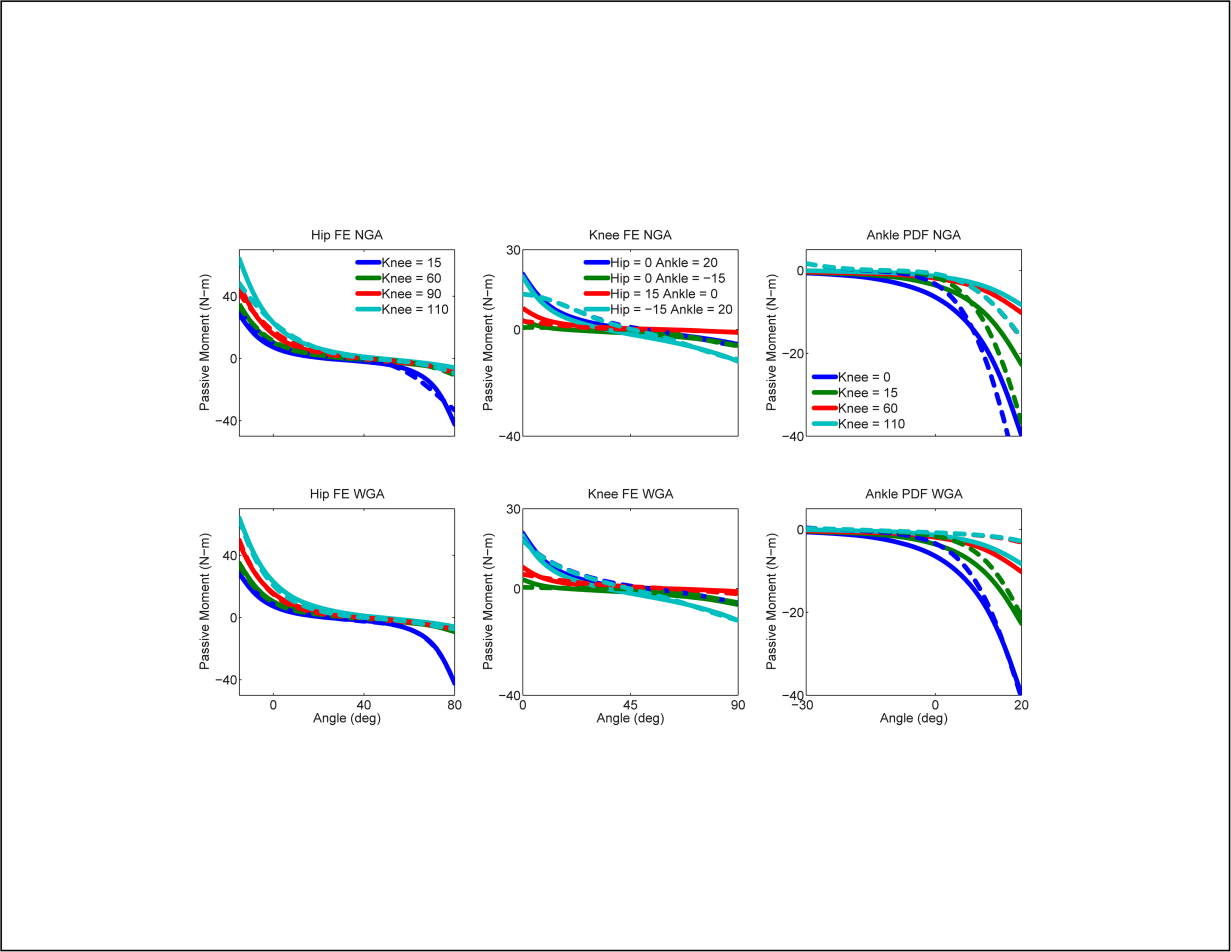
Passive joint moment matching. Passive moments predicted by our EMG-driven models calibrated using all walking speeds (dashed lines) compared to published passive moments (solid lines) for the WGA and NGA models.

Geometric adjustments improved joint moment predictions by making relatively small changes to muscle-tendon lengths and moment arms (Tables A2 and A3 in the S1 Appendix). For both “calibrate, then test” scenarios, the average change in muscle-tendon length was less than 0.9 cm (5%), while the average change in muscle moment arm was less than 0.5 cm (17%). On average, the largest muscle-tendon length change was 3.8 cm (8%) for the left semitendinosus muscle, the largest absolute mean moment arm change was 1.8 cm (44%) for the left soleus muscle about the left ankle joint, and the largest percent moment arm change was 121% (0.8 cm) for the left medial gastrocnemius muscle about the left subtalar joint. These changes allowed the WGA and NGA models to match the published passive moment curves well, though the WGA model matched them slightly better (Fig 7).

## Discussion

This study evaluated a novel method for calibrating an EMG-driven model of walking, including automated adjustment of surrogate musculoskeletal geometry, to match experimental joint moment data. In addition to geometric adjustments, the method possesses several other unique features, including scaling of EMG signals and matching of published lower extremity passive joint moment curves. The approach was evaluated using walking data collected from a hemiparetic subject, highlighting that neurological impairment may not limit the potential utility of the approach (i.e., the subject’s neural control strategy does not need to be “optimal”). When scaled generic musculoskeletal geometry was used without adjustment, the EMG-driven model was less accurate at predicting joint moments, especially for the hip. Though we cannot claim that the adjusted geometry is a more accurate representation of the subject’s actual geometry, these adjustments improved lower extremity joint moment predictions both for speeds used in the calibration process and for faster speeds omitted from calibration. When creating EMG-driven models of walking that include the hip, adjustments to musculoskeletal geometry may be especially helpful for improving the accuracy of hip moment predictions.

Our EMG-driven models with geometric adjustments predicted joint moments for walking more accurately and under more complex conditions than did previous EMG-driven studies that predicted joint moments using only walking data (Table 4) [13,15]. In our study, joint moment predictions were generated for five DOFs in both legs using 16 EMG signals per leg with a large number of walking trials collected at multiple walking speeds, including trials from faster walking speeds not included in the calibration process. In two previous EMG-driven studies that calibrated their models using only walking data, joint moments were predicted for only the ankle [13] or only the knee [15] using 7 to 10 EMG signals from a single leg with a small number of walking trials collected at a single walking speed. Despite the use of more complex conditions, our EMG-driven model still produced lower moment errors for walking speeds included in and omitted from calibration. The only EMG-driven modeling study to date to report errors in predicted hip moments during walking is Sartori et al. (2012) [14]. Though our hip moment prediction errors are much lower than those reported in that study (see Table 4), their single- and multi-DOF EMG-driven models were calibrated using data from walking plus three other activities, which likely have made their calibration process more difficult. At the same time, their high hip moment prediction errors are consistent with our findings that geometric adjustments are especially helpful for the hip.

**Table 4:** Comparison of moment error values reported in the literature with moment error values reported in this study. Other EMG-driven studies not indicated [12,16–19] have prediction errors greater than those listed in this table or use a variety of activities for calibration and/or testing and are therefore disqualified from comparison. For the knee and ankle joints, the studies shown calibrate and test their models using only gait data. Sartori et al. 2014 was the only available EMG-driven model of the hip, and was calibrated using a variety of activities.

	Literature		This Study Single-DOF		This Study Multi-DOF	
DOF	Single-DOF	Multi-DOF	NGA	WGA	NGA	WGA
Hip FE	17^1^	26^1^	4.42	3.87	6.08	4.58
Hip AA	9.7^1^	16^1^	6.39	4.02	7.56	4.41
Knee FE	7.80^2^	7.6^1^	4.61	4.27	5.70	4.76
Ankle PDF	6.03^3^	16^1^	6.51	4.70	7.37	5.56
Ankle IE	—	—	2.96	2.36	4.53	3.62

^1^Sartori *et al*. [14] MAE (values estimated from figures since values for walking only were not explicitly stated)

^2^Kumar *et al*. [15] root mean square error (RMSE)

^3^Bogey *et al*. [13] RMSE

Most studies calibrate their EMG-driven models to predict moments about a single DOF, which is a simpler problem than predicting moments about five DOFs simultaneously. Sartori *et al*. (2012) [14] found that single-DOF NGA models calibrated with similar accuracy as a four-DOF NGA model. In contrast, when we calibrated single-DOF NGA and WGA models using our optimization framework, moment errors were always lower than with the corresponding multi-DOF model (Table 4). This finding makes sense since multi-DOF models constrain the solution more than do single-DOF models due to inter-joint coupling caused by muscles that actuate multiple DOFs. Interestingly, our multi-DOF WGA model produced comparable moment errors (sometimes slightly better, sometimes slightly worse) to our single-DOF NGA models, again highlighting the value of adding geometric adjustments.

In addition to adjustment of surrogate musculoskeletal geometry, our EMG-driven modeling approach possessed six other unique features that likely improved our moment predictions even without geometric adjustments. First, our study utilized fine-wire EMG data from several deep muscles. Fine-wire EMG data allowed us to include potentially important muscles omitted from most other studies: iliopsoas, tibialis posterior, flexor digitorum longus, and extensor digitorum longus. Omission of these muscles likely contributed to increased moment prediction errors in previous studies, especially omission of iliopsoas for the hip flexion moment. Secondly, our study filtered EMG data with a variable low pass cutoff frequency that depended on the period of the gait cycle. When using a constant low pass cutoff frequency, we found that slow gait speeds would have comparatively noisier EMG signals than did faster speeds, which adversely affected our moment predictions. In contrast, when a variable low pass cutoff frequency was used, moment predictions became more reliable across speeds. Third, our study optimized scale factors defining maximum EMG values. Most studies normalize EMG data to a maximum voluntary contraction trial (MVC) or the maximum EMG value over all collected trials. However, these methods may be unreliable indicators of maximum muscle excitation [53], and true MVC trials are often hard to obtain. Furthermore, maximal M-wave measurements demonstrate that MVC trials produce EMG values that are smaller than maximum EMG [53–55]. Therefore, we decided to optimize a muscle excitation scale factor and penalize it for deviating away from its initial value. Inclusion of optimized excitation scale factors was one of the most valuable unique additions in our approach. Fourth, our study included matching of experimentally measured passive joint moments reported in the literature [51]. These moments corresponded to much larger ranges and combinations of joint angles than occur during walking. Though these data were not subject specific, they likely helped the muscles in our model to traverse reasonable ranges on their normalized force-length curves. Matching of passive joint moment curves was another highly valuable unique addition in our approach. Fifth, our study used a larger number of walking trials for model calibration and testing. Use of a large number of trials allowed us to minimize the impact of outlier trials in both our calibration and testing process. It also allowed us to capture the broadest possible variability in the subject’s walking data, which was important since our method uses only walking data for calibration. Lastly, our study included kinematic calibration of lower extremity joint centers and orientations [37,48]. Previous studies have demonstrated that inverse dynamics moments are sensitive to the position and orientation of joint centers in the body segments [56]. As a result, the moments being matched during calibration may not be the true moments produced by muscles, resulting in EMG-driven calibration and prediction errors. Furthermore, placing a joint center in the wrong location causes offsets in muscle moment arms, further decreasing the quality of the moment predictions. Calibration of lower limb joint positions and orientations may have eliminated some of these modeling errors, thereby improving EMG-driven predictions.

While adjustments to geometric parameter values greatly reduced moment prediction errors, the accuracy with which the adjusted geometry represents the subject is unknown. Scaled generic models can have errors in mean moment arm values on the order of 3 to 4 cm [30]. Similarly, errors in muscle-tendon lengths can be 10 cm or more compared to geometric data obtained from MR images [30]. Such errors have been shown to have a significant impact on predicted joint moments in an EMG-driven knee model [29]. In our study, the largest average moment arm change was 1.8 cm, while the largest average muscle-tendon length change was 3.8 cm (Tables A2 and A3). These changes are well within the error ranges reported in the literature, suggesting that the geometric adjustments were at least reasonable.

While other studies have used varied movements and dynamometer data to calibrate and test their EMG-driven models, we purposefully used only walking data combined with published passive joint moment data for our calibration process. Restoring normal walking function is a common and important clinical goal. Therefore, models that can reproduce experimental walking data have an increased likelihood of being clinically useful. Furthermore, it could be difficult in a time-limited clinical setting with function-limited patients to collect EMG, motion capture, and ground reaction data for a wide range of movement tasks. For these reasons, we decided to calibrate our EMG-driven models using only the subject’s walking data and published passive joint moment data.

We made several decisions to account for the limitations of using primarily walking data for model calibration. To increase the information content in our calibration data, we used a large number of walking trials (10 per speed for either three or five speeds). As indicated by post-hoc statistical analyses, this approach resulted in joint angles, joint moments, and EMG amplitudes that were statistically different between the faster and slower walking speeds. Since walking data provide information over only limited ranges of joint motion and loading, we included published passive joint moment data [51] in our calibration process. This decision provided moment calibration information over broader ranges of motion than occur during walking. While our hemiparetic subject is likely to be less flexible than the healthy subjects used in [51], these unique data still represent the general trends in passive moments one might expect to observe in any ambulatory individual. Without including these extra data, the passive moments predicted at extreme joint angles outside the bounds of walking were unrealistic, with muscles generating passive forces that were well above maximum isometric force. Nonetheless, since our EMG-driven model calibration process was based primarily on walking data, it may not predict joint moments well for motions other than walking.

The ability of our EMG-driven model with geometric adjustments to predict joint moments well for faster non-calibration walking speeds may make this model clinically useful for predictive gait optimization studies. By incorporating our EMG-driven model into a dynamic patient-specific full-body walking model that includes deformable foot-ground contact models, researchers could predict how changes in a patient’s muscle excitations could alter the patient’s gait pattern in a favorable way [57]. Muscle excitations could be controlled individually or coupled together through muscle synergies calculated from the patient’s EMG data [57]. For a subject with hemiparesis, the optimizations could seek to identify minimal changes in the patient’s muscle excitations that would produce a desired improvement in walking speed and bilateral symmetry. The predicted neural control and gait pattern changes could potentially help clinicians determine which muscles should be targeted for excitation timing changes, strength increases, and/or functional electrical stimulation (i.e., treatment prescription), as well as how much of each type of change is required (i.e., treatment dosage).

For such approach to become clinically useful, computational speed will be an important consideration. For all 5 speeds together with 10 gait trials per speed, EMG-driven model calibration performed using 10 of 12 cores on a 2 GHz Intel Xeon workstation required approximately 2 hours of CPU time for the NGA approach and 10 hours for the WGA approach. The NGA approach has roughly a third of the design variables of the WGA approach and skips one round of step-wise optimizations. For both approaches, repeated spline sampling of processed EMG data to accommodate eletromechanical delays is the primary computational bottleneck. While the WGA CPU time in particular may seem high, it needs to be viewed in light of the larger “computational neurorehabilitation” treatment design process. It currently takes about half a day to collect the necessary walking data and one to three days to process it before an EMG-driven model can be calibrated. It also takes about day to calibrate a full-body walking model to match the subject’s EMG, marker motion, and ground reaction data simultaneously with a dynamically consistent model. Once the full-body model is calibrated, however, new walking motions can be predicted via direct collocation optimal control in about 30 minutes of CPU time [57]. Thus, it would currently be impossible to collect data, process it, calibrate the EMG-driven model, calibrate the dynamic full-body walking model, and generate new walking motion predictions within a single clinical visit. Nonetheless, development of personalized neurorehabilitation prescriptions off-line could still be valuable if the prescriptions are more effective and efficient than those currently developed solely through clinical intuition.

One of the primary limitations of the present study was the use of a deterministic rather than stochastic EMG-driven model development and evaluation process. When undertaking this study, our goal was to build upon published EMG-driven modeling studies [12,14–16,28] by adding one primary enhancement (adjustment of parameter values related to surrogate representations of the musculoskeletal geometry) along with several secondary enhancements (matching of published passive joint moment data, adjusting EMG normalization parameters, calibrating joint positions/orientations to improve the accuracy of inverse dynamic joint moments). These previous studies followed a similar deterministic model development and evaluation approach. In contrast, a stochastic model development and evaluation approach would account for how uncertainties in experimental inputs (i.e., ground reactions, marker motions) and skeletal model parameter values (i.e., joint positions and orientations, segment mass properties) affect the inverse dynamic joint moments being matched in the model calibration process. Such an approach, which would need to be designed and implemented differently than the present deterministic approach, would facilitate assessment of EMG-driven model joint moment predictions in light of the amount of uncertainty present in the net joint moments from inverse dynamics.

To explore whether inverse dynamic joint moment uncertainties would have affected our findings significantly, we performed a post-hoc Monte Carlo analysis (see S1 Appendix for details) on a representative walking trial from the 0.8 m/s speed, where inverse dynamic joint moment errors would be expected to be the largest. The analysis performed 2000 perturbed inverse dynamic analyses, where each iteration added estimated uncertainties to the input ground reactions, marker motions, and skeletal model joint positions/orientations (Table A4 in S1 Appendix). For each iteration, mean absolute error (MAE) across the gait cycle was calculated for each inverse dynamic moment, and the mean and standard deviation of the 2000 MAE values were then calculated (Table A5 in S1 Appendix). Compared to these mean MAE values, the mean MAE values calculated for the NGA and WGA methods were between 1.7 and 9.4 times larger (Table A5 in S1 Appendix), indicating that both EMG-driven modeling methods were fitting data rather than noise and that improvements from the WGA method over the NGA method reported in Tables 2 and 3 were likely true improvements.

This study possesses several other important limitations that have not been mentioned previously and should be considered when interpreting our results. First, due to the complexity of the EMG-driven model development process, we have only modeled a single hemiparetic subject thus far. However, the purpose of the present study was to evaluate the feasibility and potential benefits of our proposed EMG-driven modeling method with geometric adjustments, and analysis of a single subject is sufficient for those purposes. Second, our method requires EMG data, including signals from deep muscles acquired with fine-wire electrodes, for all muscles that contribute significantly to the task being modeled. In our case, this requirement meant that fine-wire data were needed from iliopsoas in particular. Without prior knowledge of muscle excitation patterns, our optimization problem would be highly underdetermined with no well-defined solution. For studies lacking critical EMG data, geometric adjustments using the methods described here would be difficult. Third, our model includes muscles for which EMG data are not available. For these muscles, we apply excitations from anatomically related muscles (review Table 1 describing how 16 EMG signals were applied to 35 muscles per leg), which may not accurately represent the true excitations. Fourth, our method used initial model parameter values and bounds taken from the literature. There is no guarantee that literature values will represent well the anatomy of a particular subject, and even using them for bounds may over-constrain the model. Unfortunately, clinical measurement of patient-specific model parameter values is not currently possible, and thus literature values must suffice as a starting point for the time being. Fifth, we assumed bilateral symmetry for most model parameter values, despite the fact that our subject had suffered a stroke. We evaluated this assumption by removing the bilateral symmetry requirement and recalibrating each leg separately across all speeds. While this modification produced small improvements in joint moment predictions, the optimizations were more likely to get stuck in a local minimum. Furthermore, computation time increased significantly due to a near doubling in the number of model parameter values. For these reasons, we maintained bilateral symmetry for all model parameter values except excitation scale factors and time delays. For subjects with greater neurological impairment, a bilateral symmetry assumption may be more limiting.

In conclusion, the novel EMG-driven model calibration method with geometric adjustments presented in this study improved joint moment prediction accuracy for walking compared to results generated using a scaled geometric musculoskeletal model. The proposed EMG-driven model creation process can be almost entirely automated and requires little effort when compared with construction of complex geometric models from MR and/or CT data. Because of its improved moment prediction accuracy, our modeling method with geometric adjustments may prove useful in future clinical applications. Based on the results of this study, we recommend that researchers incorporate geometric adjustments into their EMG-driven modeling process to improve the accuracy of joint moment predictions for walking, especially at the hip.

## Supporting information

S1 Appendix Appendix of supporting information. This information includes details of optimization problem formulation, details of outlier identification methods, tables of muscle-tendon length and moment arm changes, and estimation of inverse dynamic joint moment errors via Monte Carlo analyses.
(PDF)
