# Supplementary material for: Lower extremity EMG-driven modeling of walking with automated adjustment of musculoskeletal geometry
Source: PLoS One. 2017 Jul 11;12(7):e0179698. doi: 10.1371/journal.pone.0179698 (PMC5507406; doi:10.1371/journal.pone.0179698)
Supplement: S1 Appendix — This information includes details of optimization problem formulation, details of outlier identification methods, tables of muscle-tendon length and moment arm changes, and estimation of inverse dynamic joint moment errors via Monte Carlo analyses. (PDF) [file pone.0179698.s001.pdf]

S1 Appendix

Lower Extremity EMG-driven Modeling of Walking with Automated Adjustment of Musculoskeletal Geometry

Andrew J. Meyer[1], Carolynn Patten[2,3], and Benjamin J. Fregly[1]

[1] Department of Mechanical & Aerospace Engineering, University of Florida, Gainesville, FL

[2]Department of Physical Therapy, University of Florida, Gainesville, FL

[3]Neural Control of Movement Lab, Malcom Randall VA Medical Center, Gainesville, FL

Details of Optimization Problem Formulation

Calibration of our EMG-driven model was achieved by performing a nonlinear constrained optimization. Initial guesses, bounds, and cost function terms used in the optimization (Table 2) were specified such that the final solution would remain anatomically realistic and close to the initial model. In addition to minimizing errors in walking and passive joint moment curves, the cost function included penalty terms that acted as "soft constraints" to limit deviations of model parameter values and curves away from the initial model. Inequality "hard constraints" were also included in the problem formulation to keep normalized muscle lengths and velocities within known realistic ranges and to prevent muscle moment arms from switching signs. For each type of parameter or curve with a specified reference value and associated allowable deviation (see Table A1), a single cost function penalty term was formulated by calculating deviations of the model parameters or curves from the reference value, normalizing all deviations by the allowable deviation (which could be exceeded), and calculating the mean squared

value. For example, for shape changes in muscle-tendon length curves, there were 35 muscles per leg x 2 legs x 101 time points per gait cycle x 50 gait cycles = 530,250 shape deviation values calculated relative to reference curves from the initial model. The corresponding penalty term in the cost function was calculated by dividing each deviation value by 0.25, squaring each normalized deviation, taking the sum, and finally dividing by 530,250.

Table A1: Initial guesses, bounds, reference values, and allowable deviations for constant model parameters (top half) and time-varying model curves (bottom half). For quantities with a specified Allowable Deviation, cost function penalty terms were calculated by finding the deviation of the model parameter or curve from the specified reference value, normalizing by the allowable deviation, and calculating the mean squared value. For quantities without a specified Allowable Deviation, bounds were enforced using nonlinear inequality constraints. Surrogate geometry curves refer to muscle-tendon lengths and moment arms but not velocities. Allowable deviation values were chosen iteratively to ensure that parameter values did not change far from the reference values. Different allowable deviations were used for the hip, knee, and ankle surrogate geometry shape changes.

Model Parameter (top) or Curve (bottom)	Initial Guess	Lower Bound	Upper Bound	Reference Value	Allowable Deviation
**Normalized EMG scale factor**	0.25	0.05	1	1	0.5
**Electromechanical delay (ms)**	50	0	100	—	—
**Activation time constant (ms)**	15	5	35	15	15
**Nonlinearity constant**	0	0	0.35	0	0.25
**Optimal muscle fiber length scale factor**	1	0.75	1.25	1	0.15 [4]
**Tendon slack length scale factor**	1	0.75	1.25	1	0.15 [4]
**Normalized EMG scale factor variations**	—	—	—	0	1
**Electromechanical delay variations (ms)**	—	—	—	0	100 [4]
**Normalized muscle length**	—	0.3	1.3	—	—
**Maximum normalized muscle length**	—	0.8	—	—	—
**Minimum normalized muscle length**	—	—	1.0	—	—
**Normalized muscle velocity**	—	-1	1	—	—
**Surrogate geometry mean value changes**	—	—	—	0	0.5 [5]
**Surrogate geometry shape changes**	—	—	—	0	0.25 or 0.125 [5]
**Normalized muscle length variations**	—	—	—	0	1 [5]
**Muscle moment arm variations (cm)**	—	—	—	0	2 [5]
**Inverse dynamic joint moment errors**	—	—	—	0	1
**Passive joint moment errors**	—	—	—	0	1

Additional explanation is helpful for understanding how and why some of the design variables and their initial values and bounds were selected. Normalized processed EMG signals were multiplied by an additional scale factor to account for the fact that the maximum processed EMG value for each muscle was unlikely to be the true maximum. This decision is supported by experimental data demonstrating that the maximal M-wave is significantly larger than volitional EMG measured during walking or maximum voluntary contraction [1–3]. The upper bound for electromechanical delay was based on

values reported in the literature [4]. The initial guess of 0 for activation nonlinearity constants represents a linear relationship between neural activation and muscle activation. For surrogate geometry mean value changes (i.e., changes in mean value of a muscle-tendon length or moment arm curve), deviation was calculated as the difference in mean values between adjusted and reference curves divided by the maximum absolute value of the reference curve. For surrogate geometry shape changes (i.e., change in shape of a muscle-tendon length or moment arm curve), deviation at each time point was calculated as the difference between demeaned adjusted and reference curves divided by the range of the reference curve. For moment arm shape changes, hip moment arm deviations were normalized by 0.25 while knee and ankle moment arm deviations were normalized by 0.125 based on uncertainties reported in literature [5]. All parameter values were the same for both legs with the exception of electromechanical delays and EMG scale factors, which were specific to each muscle in each leg since the subject was hemiparetic.

Muscles that function within a physiological group or share a common EMG signal were given special treatment in the optimization cost function. For these muscles, additional "soft constraints" were included in the cost function to ensure that curves or parameter values from related muscles remained similar. For normalized EMG scale factor variations and electromechanical delay variations, deviation was calculated as the standard deviation of the scale factors or delays for all muscles in the group. For normalized muscle length variations and moment arm variations, deviation at each time point was calculated as the standard deviation of the lengths or moments arms for all muscles in the group.

Determination of appropriate initial values for optimal muscle fiber lengths and tendon slack lengths required additional steps. Initial values taken from the literature [6] produced excessively high or low normalized muscle lengths for some muscles. Excessively high normalized muscle lengths produce unrealistically high passive joint moments, while excessively low normalized muscle lengths cause

muscles to produce little force even when fully activated. To address this issue, we performed a preliminary optimization to determine initial values of optimal muscle fiber lengths and tendon slack lengths that placed every muscle within a physiological operating range over the gait cycle [7]. The optimization cost function minimized changes in initial parameter values taken from the literature while also minimizing errors in model-predicted passive joint moments relative to experimental curves reported in the literature [8]. Exponential terms were also included in the cost function to limit normalized muscle lengths during walking to between 0.3 and 1.3 [7]. The pre-optimized optimal muscle fiber length and tendon slack length values were used in the larger EMG-driven calibration process.

Three categories of model parameter values were unaltered during the calibration process. For the activation model, the deactivation time constant for each muscle was specified to be four times the muscle's activation time constant. For the Hill-type muscle model, peak isometric force were calculated using regression equations reported in literature [9], with a muscle specific tension of 61 N/cm^2 [6]. Pennation angles were taken from the initial model [6]. Since muscle excitations were scaled during the EMG-driven model calibration process, adjustment of peak isometric force and pennation angle values would have been redundant since all three quantities scale the muscle force in the Hill-type model equations.

Details of Outlier Identification Methods

Given the curves output by OpenSim analyses, we performed a series of tests to identify and remove outlier trials and select trials for use in calibration and testing. First, for trials of the same speed, any gait cycle where a muscle's peak EMG value was greater than three times its median peak value was removed from the data set. Next, for each walking speed, any trial with a joint moment, angle, angular

velocity, or moment arm curve more than five standard deviations away from its mean curve at any point in the gait cycle was removed from the data set. Once outlier trials were identified and removed, calibration and testing trials were selected from the remaining trials. For each gait speed, 20 of the remaining trials were selected for subsequent analysis - ten for calibration and ten for testing.

Tables of Muscle-tendon Length and Moment Arm Changes

The tables below provide quantitative information on how muscle-tendon lengths and moment arms were changed for the EMG-drive model calibration process with geometric adjustments when data from all five walking speeds were used for calibration (Table A2) and when data from only the three slowest walking speeds were used for calibration (Table A3).

Table A2: Mean absolute changes in muscle-tendon lengths and moment arms for calibration walking trials from a model calibration performed using data from all walking speeds. Percent changes were calculated using each curve's maximum absolute value.

	ℓ^{MT}		Hip FE		Hip AA		Knee FE		Ankle PDF		Ankle IE	
Muscle	cm	%	cm	%	cm	%	cm	%	cm	%	cm	%
Adductor brevis	2.29	15.32	0.06	1.50	0.06	0.77	--	--	--	--	--	--
Adductor longus	0.34	1.38	0.86	14.33	1.91	24.19	--	--	--	--	--	--
Adductor magnus distal	1.79	7.14	0.48	9.25	0.05	0.89	--	--	--	--	--	--
Adductor magnus ischial	1.68	4.54	0.05	0.71	0.11	2.25	--	--	--	--	--	--
Adductor magnus middle	1.09	6.79	0.77	27.96	0.14	2.04	--	--	--	--	--	--
Adductor magnus proximal	1.17	9.36	0.19	6.84	0.07	1.15	--	--	--	--	--	--
Gluteus maximus superior	1.35	5.80	0.29	5.08	0.69	22.27	--	--	--	--	--	--
Gluteus maximus middle	0.88	3.28	0.67	9.51	0.56	24.23	--	--	--	--	--	--
Gluteus maximus inferior	0.87	3.02	0.83	9.25	0.88	17.71	--	--	--	--	--	--
Gluteus medius anterior	0.42	3.15	0.99	30.76	1.66	35.65	--	--	--	--	--	--
Gluteus medius middle	0.65	4.44	0.69	17.72	0.46	10.65	--	--	--	--	--	--
Gluteus medius posterior	0.83	6.28	0.73	19.66	0.87	21.81	--	--	--	--	--	--
Gluteus minimus anterior	1.38	15.62	0.34	16.76	0.24	5.39	--	--	--	--	--	--
Gluteus minimus middle	1.77	20.16	1.06	42.91	0.21	4.75	--	--	--	--	--	--
Gluteus minimus posterior	0.59	6.15	0.67	23.82	0.11	2.68	--	--	--	--	--	--
Iliacus	2.19	9.73	0.31	6.94	0.75	75.35	--	--	--	--	--	--
Psoas	1.88	7.90	0.38	11.32	0.25	19.30	--	--	--	--	--	--
Semimembranosus	0.64	1.52	0.43	6.72	0.47	20.58	0.41	8.32	--	--	--	--

Semitendinosus	3.22	6.84	1.14	15.08	1.06	29.37	0.41	6.94	--	--	--	--
Biceps femoris long head	0.62	1.45	0.18	2.53	1.71	54.45	0.55	13.54	--	--	--	--
Biceps femoris short head	1.02	4.47	--	--	--	--	0.20	4.91	--	--	--	--
Rectus femoris	0.85	2.00	0.93	16.59	1.68	71.71	0.77	16.44	--	--	--	--
Vastus medialis	0.11	0.51	--	--	--	--	0.27	6.38	--	--	--	--
Vastus lateralis	0.98	4.13	--	--	--	--	0.34	8.00	--	--	--	--
Vastus intermedius	0.87	3.92	--	--	--	--	0.36	8.43	--	--	--	--
Lateral gastrocnemius	0.22	0.44	--	--	--	--	0.34	13.43	0.43	9.74	0.23	26.59
Medial gastrocnemius	0.26	0.51	--	--	--	--	0.28	9.23	0.43	9.95	0.60	87.01
Tibialis anterior	0.25	0.76	--	--	--	--	--	--	0.05	0.99	0.51	41.96
Tibialis posterior	0.02	0.05	--	--	--	--	--	--	0.22	24.20	0.12	6.60
Peroneus tertius	0.41	1.90	--	--	--	--	--	--	0.48	67.94	0.28	9.38
Peroneus longus	0.10	0.25	--	--	--	--	--	--	0.16	16.56	0.13	4.81
Peroneus tertius	0.19	1.01	--	--	--	--	--	--	0.19	6.11	0.67	27.73
Soleus	0.15	0.41	--	--	--	--	--	--	1.68	40.06	0.17	21.38
Extensor digitorum longus	0.04	0.09	--	--	--	--	--	--	0.07	1.77	0.05	3.19
Flexor digitorum longus	0.02	0.04	--	--	--	--	--	--	0.01	0.92	0.12	7.65
Mean Absolute Change	0.89	4.58	0.57	14.06	0.66	21.29	0.39	9.56	0.37	17.82	0.29	23.63
Max Absolute Change	3.22	20.16	1.14	42.91	1.91	75.35	0.77	16.44	1.68	67.94	0.67	87.01

Table A3: Mean absolute changes in muscle-tendon lengths and moment arms for calibration walking trials from a model calibration performed using data from only the three slowest speeds. Percent changes were calculated with respect to each curve's maximum absolute value.

	ℓ^{MT}		Hip FE		Hip AA		Knee FE		Ankle PDF		Ankle IE	
Muscle	cm	%	cm	%	cm	%	cm	%	cm	%	cm	%
Adductor brevis	2.48	16.69	0.04	1.05	0.07	1.01	--	--	--	--	--	--
Adductor longus	1.12	4.60	0.14	2.32	0.19	2.43	--	--	--	--	--	--
Adductor magnus distal	1.20	4.88	0.35	6.88	0.19	3.34	--	--	--	--	--	--
Adductor magnus ischial	0.64	1.76	0.17	2.51	0.09	1.86	--	--	--	--	--	--
Adductor magnus middle	1.42	8.88	0.41	17.10	0.05	0.80	--	--	--	--	--	--
Adductor magnus proximal	0.59	4.73	1.70	60.79	1.07	17.56	--	--	--	--	--	--
Gluteus maximus superior	1.10	4.79	0.13	2.20	0.51	17.36	--	--	--	--	--	--
Gluteus maximus middle	0.82	3.13	0.40	5.67	0.44	21.33	--	--	--	--	--	--
Gluteus maximus inferior	0.53	1.90	0.40	4.45	0.55	11.34	--	--	--	--	--	--
Gluteus medius anterior	1.56	11.79	1.02	32.04	0.89	18.97	--	--	--	--	--	--
Gluteus medius middle	0.26	1.79	0.52	13.77	0.48	11.30	--	--	--	--	--	--
Gluteus medius posterior	0.19	1.43	0.49	13.58	1.21	30.87	--	--	--	--	--	--
Gluteus minimus anterior	0.72	8.18	0.46	23.39	0.20	4.45	--	--	--	--	--	--
Gluteus minimus middle	0.58	6.55	0.56	23.71	0.13	2.85	--	--	--	--	--	--
Gluteus minimus posterior	0.56	5.80	0.60	21.91	0.11	2.55	--	--	--	--	--	--
Iliacus	1.49	6.71	0.26	5.85	0.60	79.19	--	--	--	--	--	--
Psoas	1.42	5.98	0.09	2.85	0.28	25.29	--	--	--	--	--	--
Semimembranosus	0.80	1.93	0.41	6.38	0.33	14.94	0.50	10.10	--	--	--	--

Semitendinosus	3.80	8.21	0.56	7.38	0.83	23.58	0.32	5.41	--	--	--	--
Biceps femoris long head	0.37	0.88	0.26	3.63	0.80	25.93	0.67	16.63	--	--	--	--
Biceps femoris short head	0.91	4.02	--	--	--	--	0.12	3.08	--	--	--	--
Rectus femoris	0.81	1.93	0.89	16.06	0.98	42.92	0.68	14.49	--	--	--	--
Vastus medialis	0.69	3.31	--	--	--	--	0.20	4.71	--	--	--	--
Vastus lateralis	1.58	6.72	--	--	--	--	0.25	5.90	--	--	--	--
Vastus intermedius	1.20	5.44	--	--	--	--	0.31	7.11	--	--	--	--
Lateral gastrocnemius	0.24	0.48	--	--	--	--	0.31	12.20	0.76	17.58	0.61	69.60
Medial gastrocnemius	0.30	0.59	--	--	--	--	0.91	30.36	0.61	14.37	0.81	116.71
Tibialis anterior	0.19	0.57	--	--	--	--	--	--	0.05	1.17	0.37	29.89
Tibialis posterior	0.02	0.04	--	--	--	--	--	--	0.20	21.45	0.11	6.18
Peroneus tertius	0.20	0.94	--	--	--	--	--	--	0.36	51.01	0.19	6.48
Peroneus longus	0.08	0.19	--	--	--	--	--	--	0.09	9.04	0.08	2.92
Peroneus tertius	0.22	1.13	--	--	--	--	--	--	0.15	4.86	0.75	30.95
Soleus	0.12	0.34	--	--	--	--	--	--	1.50	36.24	0.13	17.21
Extensor digitorum longus	0.01	0.02	--	--	--	--	--	--	0.07	1.82	0.09	6.05
Flexor digitorum longus	0.02	0.05	--	--	--	--	--	--	0.06	8.01	0.04	2.32
Mean Absolute Change	0.81	3.90	0.47	13.03	0.48	17.14	0.43	11.00	0.39	16.55	0.32	28.83
Max Absolute Change	3.80	16.69	1.70	60.79	1.21	79.19	0.91	30.36	1.50	51.01	0.81	116.71

Estimation of Inverse Dynamic Joint Moment Errors via Monte Carlo Analyses

To assess the sensitivity of our inverse dynamics calculations to noise in marker data and ground reaction load data as well as errors joint centers and orientations, we performed four Monte Carlo analyses that varied each of these parameters according to methods outlines in a previous study [10]. For the following simulations, the right leg and upper body segments were removed from the model to reduce computation time and also to mimic methods from a similar previous Montel Carlo study [10]. Before performing any simulations, we chose a representative gait trial of the subject walking at 0.8 m/s as the baseline for the analysis. The fastest speed of 0.8 m/s was chosen as a worst-case scenario, since the sensitivity of our inverse dynamics models was found to be greater at faster speeds. Using data from this trial, we used synthetic marker trajectories so that the model could match marker locations exactly during inverse kinematics. These trajectories were calculated by running the model through a previously

determined gait motion and outputting the marker locations from the model. With these new marker trajectories and the trial's ground reaction data, we calculated synthetic joint kinematics and moments that provided the point of comparison for the Monte Carlo analyses.

We performed a sequence of four Monte Carlo analyses using the synthetic walking data. The first Monte Carlo analysis added randomized noise signals to marker data, where a sinusoidal equation of the form $A sin(\omega t + \varphi)$, where A is the noise amplitude, ω is the frequency, and φ is the a phase shift for the noise [11]. Maximum values for each of these noise parameters are listed in Table A4. For each Monte Carlo iteration, a random value between 0 and the maximum value of each noise parameter was chosen using a uniform distribution and was added to the original signal. A different noise signal was added to each dimension of each marker trajectory. For the second Monte Carlo analysis, noise was added to the ground reactions and centers of pressure using the same model as used for marker noise. Maximum values for noise amplitude are provided in Table A4 as well. The third Monte Carlo analysis varied joint positions and orientations. Following methods detailed in a previous similar Monte Carlo study [10], 27 joint parameters were varied: 6 hip parameters, 9 knee parameters, and 12 ankle/subtalar parameters. For each Monte Carlo analysis, a new OpenSim model was created with random offsets applied to each joint parameter. These offsets were randomly determined using a uniform distribution and were allowed to vary between +/- the maximum value shown in Table A4. The fourth Monte Carlo analysis represented a worst case scenario and varied all marker data, ground reaction data, and joint parameters simultaneously. For each iteration of a Monte Carlo analysis, inverse dynamic moments were calculated with the new data/models and stored for comparison with the moments from the synthetic data. Each Monte Carlo simulation was run for 2000 iterations to achieve convergence. Joint moment curves from Monte Carlo analysis were compared with the synthetic curves, and MAE values were calculated to estimate the amount of error in inverse dynamic joint moments due to errors in experimental data and

model parameter values. Finally, these MAE errors were compared to those obtained for testing trials performed at the same speed when data from 0.8 m/s was, and was not, used in the calibration process.

Table A4: Maximum allowable changes in each of the parameter types varied in the four Monte Carlo analyses. For the marker [10-13], ground reaction (GR), and center of pressure (CoP), the max values represent the biggest possible amplitude of a sinusoidal curve that is applied to the data. For the joint centers and orientations, the max value represents a maximum possible offset to a joint definition parameter in the model.

	Max Parameter Change							
	Pelvis Markers	Thigh Markers	Shank Markers	Foot Markers	GR Load	CoP	Joint Center	Joint Orientation
	cm	cm	cm	cm	%	cm	cm	deg
Analysis 1	0.5	1	0.5	0.25	--	--	--	--
Analysis 2	--	--	--	--	1	0.5	--	--
Analysis 3	--	--	--	--	--	--	0.5	5
Analysis 4	0.5	1	0.5	0.25	1	0.5	0.5	5

From our Monte Carlo analyses, we found that the errors in inverse dynamic joint moments arriving for noise in experimental marker motion and ground reaction data and errors in joint positions and orientations were smaller than the errors in joint moment predictions produced by our NGA and WGA EMG-driven modeling methods (Table A5). When marker noise was introduced, the greatest joint moment errors were found at the hip while more distal joints had progressively smaller errors. On the other hand, joint moment errors resulting from joint position and orientation errors or ground reaction noise were more evenly distributed, with the largest errors occurring at the hip and knee for ground reaction force errors and at the knee and subtalar joint for the joint parameter errors. The analysis that varied all sources of error together produced the greatest inverse dynamic joint moment errors for all joints. The fact the MAE results from the NGA and WGA methods were generally 2 to 5 times larger than the MAE results from the worst-case Monte Carlo analysis indicate that our EMG-driven modeling methods were fitting actual data rather than noise, and furthermore, that the WGA method produces more accurate joint moment results than does the NGA method.

Table A5: Summary of mean (standard deviation in parentheses) joint moment MAE results from the four Monte Carlo analyses performed for a representative walking trial at 0.8 m/s. Comparison is provided with joint moment MAE results for the NGA and WGA methods performed on testing trials for models calibrated using (Calibration) and not using (Prediction) data from 0.8 m/s. For each Monte Carlo iteration, MAE errors were first calculated over the entire gait cycle for each joint moment. Then a mean and standard deviation were calculated for each joint moment using the 2000 individual MAE errors.

		Hip FE	Hip AA	Knee FE	Ankle FE	Ankle IE
		N-m	N-m	N-m	N-m	N-m
Monte Carlo	Marker positions	1.04 (0.37)	0.91 (0.31)	0.73 (0.24)	0.24 (0.08)	0.34 (0.11)
	Ground reactions	1.58 (0.81)	0.76 (0.35)	1.55 (0.79)	1.42 (0.70)	0.96 (0.43)
	Joint parameters	1.11 (0.52)	1.50 (0.90)	1.55 (1.00)	1.11 (0.73)	2.15 (1.41)
	All quantities	2.37 (0.81)	1.99 (0.78)	2.37 (1.01)	1.82 (0.81)	2.42 (1.37)
Calibration	NGA	7.45	8.81	4.76	7.24	5.50
	WGA	5.04	4.79	4.22	5.99	4.22
	NGA/Monte Carlo	3.14	4.43	2.01	3.97	2.27
	WGA/Monte Carlo	2.13	2.41	1.78	3.28	1.75
Prediction	NGA	7.42	9.40	6.75	6.63	5.19
	WGA	7.34	5.84	5.65	6.54	4.08
	NGA/Monte Carlo	3.13	4.72	2.84	3.63	2.15
	WGA/Monte Carlo	3.10	2.93	2.38	3.58	1.69

The fact that our Monte Carlo analyses found inverse dynamic joint moment MAEs that were smaller than our EMG-driven model joint moment MAEs is significant because it indicates that our model predictions were outside of the noise level of estimated errors in our inverse dynamic joint moment calculations. While a small amount of the improvement in our WGA results may have been due to fitting noise, most of the improvement cannot be attributed to fitting noise. The primary limitation of our Monte Carlo analyses was the use of walking data from only a single relatively slow speed (compare 0.8 m/s to a typical healthy walking speed of 1.4 m/s). Consequently, our calculated MAEs were smaller than those found in past studies. If data from faster walking speeds were used, we would expect to find larger MAEs for inverse dynamic joint moments.

References

1. Cronin NJ, Kumpulainen S, Joutjärvi T, Finni T, Piitulainen H. Spatial variability of muscle activity during human walking: The effects of different EMG normalization approaches. Neuroscience. 2015;300: 19–28. doi:10.1016/j.neuroscience.2015.05.003

2. Simonsen EB, Alkjær T, Raffalt PC. Reflex response and control of the human soleus and gastrocnemius muscles during walking and running at increasing velocity. Exp brain Res. 2012;219: 163–74. doi:10.1007/s00221-012-3075-y

3. Racinais S, Maf NA, Girard O. M-wave, h- and v-reflex recruitment curves during maximal voluntary contraction. J Clin Neurophysiol. 2013;30: 415–421.

4. Sartori M, Reggiani M, Farina D, Lloyd DG. EMG-driven forward-dynamic estimation of muscle force and joint moment about multiple degrees of freedom in the human lower extremity. PLoS One. 2012;7: e52618. doi:10.1371/journal.pone.0052618

5. Scheys L, Van Campenhout A, Spaepen A, Suetens P, Jonkers I. Personalized MR-based musculoskeletal models compared to rescaled generic models in the presence of increased femoral anteversion: effect on hip moment arm lengths. Gait Posture. 2008;28: 358–65. doi:10.1016/j.gaitpost.2008.05.002

6. Arnold EM, Ward SR, Lieber RL, Delp SL. A model of the lower limb for analysis of human movement. Ann Biomed Eng. 2010;38: 269–79. doi:10.1007/s10439-009-9852-5

7. Arnold EM, Delp SL. Fibre operating lengths of human lower limb muscles during walking. Philos Trans R Soc Lond B Biol Sci. 2011;366: 1530–9. doi:10.1098/rstb.2010.0345

8. Silder A, Whittington B, Heiderscheit B, Thelen DG. Identification of passive elastic joint moment-angle relationships in the lower extremity. J Biomech. 2007;40: 2628–35. doi:10.1016/j.jbiomech.2006.12.017

9. Handsfield GG, Meyer CH, Hart JM, Abel MF, Blemker SS. Relationships of 35 lower limb muscles to height and body mass quantified using MRI. J Biomech. 2014;47: 631–8. doi:10.1016/j.jbiomech.2013.12.002

10. Chèze, L., Fregly, B. J., and Dimnet, J. (1995). A solidification procedure to facilitate kinematic analyses based on video system data. *J. Biomech.* 28, 879–884. doi:10.1016/0021-9290(95)95278-D.

11. Lu TW, O'Connor JJ, Dortmans LJMG, Kalenak A, Croce U Della. Bone position estimation from skin marker co-ordinates using global optimisation with joint constraints. J Biomech. 1999;32: 129–34. doi:10.1016/S0021-9290(98)00158-4

12. Reinbolt JA, Schutte JF, Fregly BJ, Haftka RT, George AD, and Mitchell KH. Determination of patient-specific multi-joint kinematic models through two-level optimization. J Biomech. 2005;38:621-626. doi:10.1016/j.jbiomech.2004.03.031

13. Reinbolt JA, Haftka RT, Chmielewski TL, Fregly BJ. Are patient-specific joint and inertial parameters necessary for accurate inverse dynamics analyses of gait? IEEE Trans Biomed Eng. 2007;54: 782–793. doi:10.1109/TBME.2006.889187
